# Changes in Amino Acid and Acylcarnitine Plasma Profiles for Distinguishing Patients with Multiple Sclerosis from Healthy Controls

**DOI:** 10.1155/2020/9010937

**Published:** 2020-07-15

**Authors:** Marat F. Kasakin, Artem D. Rogachev, Elena V. Predtechenskaya, Vladimir J. Zaigraev, Vladimir V. Koval, Andrey G. Pokrovsky

**Affiliations:** ^1^Institute of Chemical Biology and Fundamental Medicine, The Siberian Branch of the Russian Academy of Sciences, Novosibirsk, Russia; ^2^Novosibirsk Institute of Organic Chemistry, The Siberian Branch of the Russian Academy of Sciences, Novosibirsk, Russia; ^3^Novosibirsk State University, Novosibirsk, Russia

## Abstract

McDonald criteria and magnetic resonance imaging (MRI) are used for the diagnosis of multiple sclerosis (MS); nevertheless, it takes a considerable amount of time to make a clinical decision. Amino acid and fatty acid metabolic pathways are disturbed in MS, and this information could be useful for diagnosis. The aim of our study was to find changes in amino acid and acylcarnitine plasma profiles for distinguishing patients with multiple sclerosis from healthy controls. We have applied a targeted metabolomics approach based on tandem mass-spectrometric analysis of amino acids and acylcarnitines in dried plasma spots followed by multivariate statistical analysis for discovery of differences between MS (*n* = 16) and control (*n* = 12) groups. It was found that partial least square discriminant analysis yielded better group classification as compared to principal component linear discriminant analysis and the random forest algorithm. All the three models detected noticeable changes in the amino acid and acylcarnitine profiles in the MS group relative to the control group. Our results hold promise for further development of the clinical decision support system.

## 1. Introduction

Multiple sclerosis (MS) is one of the autoimmune disorders causing demyelination of axons [1, 2]. Modern diagnosis of MS is based on the revised McDonald criteria including magnetic resonance imaging (MRI) to confirm the result [3]. Even though many risk factors for MS have been established [4], it is unclear whether MS will progress after the first clinical symptoms or will be followed by remission [5]. The development of new methods for the diagnosis and prognosis of MS is a highly relevant research topic.

Metabolomics is a powerful approach for the discovery of biomarkers and investigation of the pathogenesis of human diseases [6, 7]. Multivariate statistical analysis is frequently applied to a whole preprocessed metabolomics dataset in metabolomics studies of human diseases, particularly MS [8, 9]. Predictive models involving several statistically significant markers outperform a single-marker model in terms of area under the curve (AUC) metrics and distinguish multiple groups with partially shared markers among them [10]. The advantage of metabolomic profiling was used to separate clinical groups into subgroups, particularly to distinguish the relapsing-remitting type and secondary progressive type of MS [11].

Amino acid and fatty acid metabolic pathways are known to be disturbed in MS [12–15]. Thus, glutamate toxicity is linked with demyelination and other pathophysiological processes in MS [16, 17]. Several amino acids have been proposed to be potential biomarkers of MS in different biological samples: methionine in serum [18]; phenylalanine in cerebrospinal fluid (CSF) [10]; leucine, asparagine, ornithine, glutamine, and glutamate in plasma [19]; and amino acid derivatives in urine [20]. Glutamate was validated as a substantial biomarker for classification of MS and other neurological diseases in a study on amino acid and acylcarnitine profiles in CSF [21]. Acylcarnitines also play an important role in energy metabolism by participating in the transfer of fatty acids into mitochondria [22]. These data suggest that acylcarnitines are an interesting object for research into biomarkers of MS.

The main aim of this study was to find possible differences in amino acid and acylcarnitine profiles in plasma between healthy controls and an MS group by means of multivariate analysis algorithms and to compare predictive effectiveness of the models at classifying the healthy group and MS group. The second aim was to identify potential biomarkers of MS among amino acids and acylcarnitines. The proposed study design may be useful for high-throughput and robust sample preparation and analysis and offers an opportunity to scale this analysis up to large cohorts in future studies.

## 2. Methods

### 2.1. Patients and Collection of Plasma Samples

We recruited 16 patients with MS (14 with relapsing-remitting MS and two with secondary progressive MS, all women) at the Department of Neurology of the 2nd Novosibirsk Emergency Hospital according to the McDonald criteria. The control group (12 non-MS subjects) was formed from women of the same age band as the MS group (Table 1). Fasting blood samples were collected into 4 ml BD Vacutainer® Heparin tubes with 68 IU of lithium heparinate. Plasma was separated via centrifugation at 2000 × *g* for 15 min, then immediately frozen and stored at -70°С until sample preparation. The study was conducted according to the Code of Ethics of the World Medical Association (Declaration of Helsinki).

### 2.2. Sample Preparation and Analysis

Plasma samples were thawed at room temperature, and 20 *μ*l aliquots of samples were spotted onto Whatman 903 Protein Saver cards and air-dried completely. Sample preparation was performed using the MassChrom® 55000 Kit (Chromsystems, Germany) with a derivatization stage for semiquantitative liquid chromatography with mass spectrometry analysis of amino acids and acylcarnitines. Next, 3.2 mm dried plasma spot disks were punched out of the filter paper into 1.5 ml plastic tubes; then, 200 *μ*l of an extraction solution containing internal standards was added for reconstitution of the samples. After 20 min agitation at 25°C and 600 rpm, the supernatants were transferred into new tubes and evaporated at 60°C and 600 rpm to dryness. After that, 60 *μ*l of a derivatization solution was added into the tubes and incubated for 15 min at 60°C and 600 rpm, followed by evaporation at 60°C and 600 rpm to dryness. Then, 100 *μ*l of reconstitution buffer was added to the residue and agitated until a homogeneous solution was obtained, followed by transfer into vials.

We did use dry spots of blood plasma, not whole plasma. This was done specifically to be inside the commonly accepted protocol of using dry spots of plasma.

Ten-microliter aliquots were injected into the liquid chromatography-mass spectrometry system: analysis was performed in a multiple-reaction monitoring (MRM) mode on the mass spectrometer API 3200 QTRAP (AB Sciex, USA) coupled with a chromatograph (LC-20AD Prominence, Shimadzu Corporation, Japan) without column separation.

MRM transitions and other mass spectrometry parameters are presented in the Analyst 1.6.2 (AB Sciex, USA) method acquisition report (Supplementary Data 1). Quality control samples L1 and L2 from MassCheck Amino acids and Acylcarnitines DBS control (Chromsystems, Germany) were used in this analysis.

### 2.3. Statistical Analysis

MRM data were processed in MultiQuant 2.1 Software (AB Sciex, USA), and then, the integration data was exported to a Microsoft Excel spreadsheet. Actual concentrations of metabolites were calculated according to their isotope-labeled standards. The list of metabolites and internal standards used for quantification and MRM transitions for ion detection is given in Supplementary Table S1. Subsequently, data cleaning, statistical computation, and exploratory data analysis were performed in the R software, version 3.4. All the signals above the signal-to-noise ratio of 2.0 were considered considerable for data analysis, and all metabolites that achieved signal-to-noise criteria which were met in 70% of the samples were included in data analysis. For unsupervised principal component analysis (PCA), missing data in the dataset were replaced by a mean value for the corresponding variable followed by normalizing the data variables. For supervised data analysis, missing data were replaced by the mean value for the corresponding variable in each group separately.

Unsupervised PCA was performed in R to reduce dimensionality for subsequent model building and to determine the number of principal components making the main contribution to variance.

Supervised linear discriminant analysis (LDA) was performed on principal components determined in PCA. Supervised PLS-DA was also performed for comparison as a popular alternative approach in chemometrics. The random forest (RF) algorithm was applied to scaled data without a preliminary dimension reduction to implement supervised analysis. Predictive models based on the three approaches were evaluated by the “leave one out sample” cross-validation method by means of the “caret” package. Receiver operating characteristic (ROC) curves were plotted to visualize the predictive models.

Metabolite levels were compared between the groups according to the nonparametric Mann–Whitney–Wilcoxon criteria for comparison of medians between independent groups.

### 2.4. Compliance with Ethical Standards

The ethics committee of the Institute of Chemical Biology Fundamental Medicine SB RAS (session 1-12/17) reviewed the study, and all experimental protocols were approved. Informed written consent was obtained from all recruited subjects. All procedures involving human participants were in accordance with the ethical standards of the institutional research committee and with the 1964 Helsinki Declaration and its later amendments or comparable ethical standards.

## 3. Results

### 3.1. Data Acquisition and Cleaning

Twelve plasma samples in the control group and 16 in the MS group were employed in our study. All participants were females whose age distribution between groups was controlled according to the nonparametric Mann–Whitney *U* test. A summary of the age statistics is given in Table 1.

Quantification of 43 metabolites, 13 amino acids, and 30 acylcarnitines was performed by a targeted quantitative approach with isotope-labeled internal standards. The MRM mode of data acquisition was chosen for convenient peak integration in the MultiQuant software (Supplementary Data 1). Concentrations of metabolites were calculated from the ratio of the peak area of a metabolite to its internal standard and a known concentration of the internal standard. The dataset generated and analyzed during the current study is available in the Figshare repository [23].

Injecting samples into the mass spectrometer without a prior chromatographic separation may be a big problem for acylcarnitine quantitation [24]. Some acylcarnitines are present in plasma at very low concentrations, and column separation might yield better results. In this work, we did not use chromatographic separation because we created a fast screening method for metabolite determination.

Data cleaning criteria such as the signal-to-noise ratio, threshold for complete cases, and replacement of missing values of the “NA” type were determined during exploratory data analysis in R. Our objective at this stage was to save as many variables as possible for further data analysis and at the same time to preserve the number of observations in small groups. Consequently, all the variables involving more than 30% of the missing values were removed from the dataset. The remaining missing values under the threshold were replaced by the mean of the variable of all observations followed by unsupervised PCA. In the case of supervised analysis, all the variables in each group of observations containing more than 30% of the missing values were removed, and the remaining missing values in each group were replaced by the mean value of the variable in this group. After cleaning of the data and preliminary normalization on the scale of 0 to 1 across variables, 29 metabolites were included in the unsupervised and supervised data analyses.

### 3.2. Multivariate Statistical Analysis

**Figure 1:**
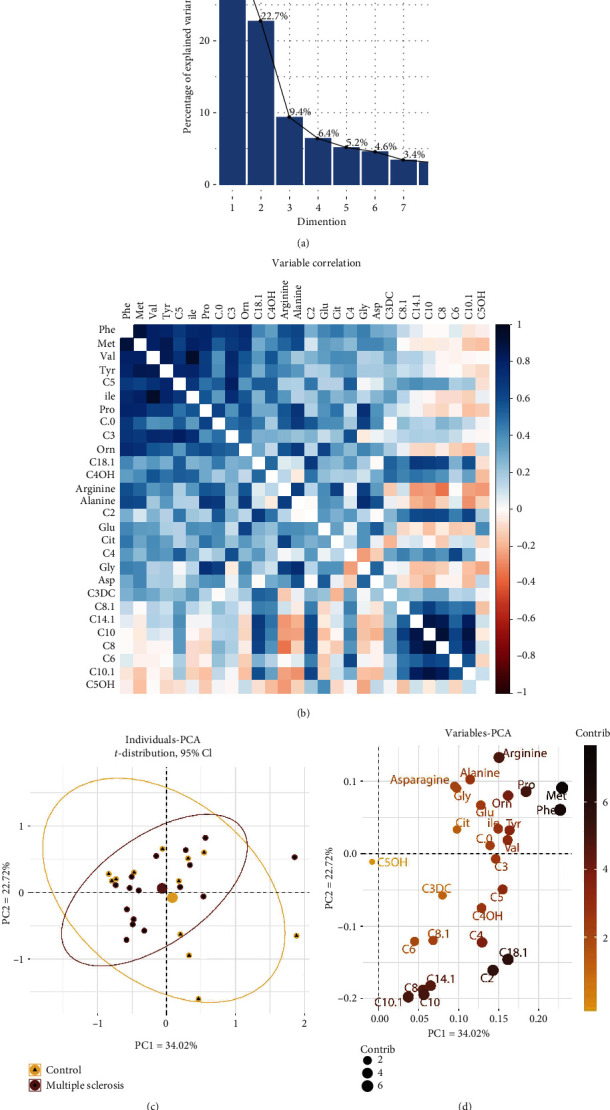
Unsupervised PCA. (a) Contribution of principal components to the data variance. (b) Correlation between variables; solid colours mean a strong correlation, and many variables correlated with each other. (c) PC1 and PC2 explain 29.91% and 20.24% of the variance, respectively, among individuals in the data. The elliptical area is equal to 95% of the probability *t*-distribution for observations in the control and MS groups. (d) The contribution of metabolites to the variance for PC1 and PC2. Met, Phe, and several acylcarnitines turned out to make the greatest contribution to the variance of the data.

Unsupervised PCA was performed for reducing dimensionality of the data and for determining the number of components making the main contribution to the data variance (Figure 1(a)). The first eight principal components were found to explain 88.8% of variance in the data. Among the variables, methionine was found to make the greatest contribution to variance followed by amino acids Phe, Pro, and Arg and acylcarnitines octadecenoyl-carnitine C18:1, acetyl-carnitine C2, decanoyl-carnitine C10, decenoyl-carnitine C10:1, tetradecenoyl-carnitine C14:1, and octanoyl-carnitine C8.

**Figure 2:**
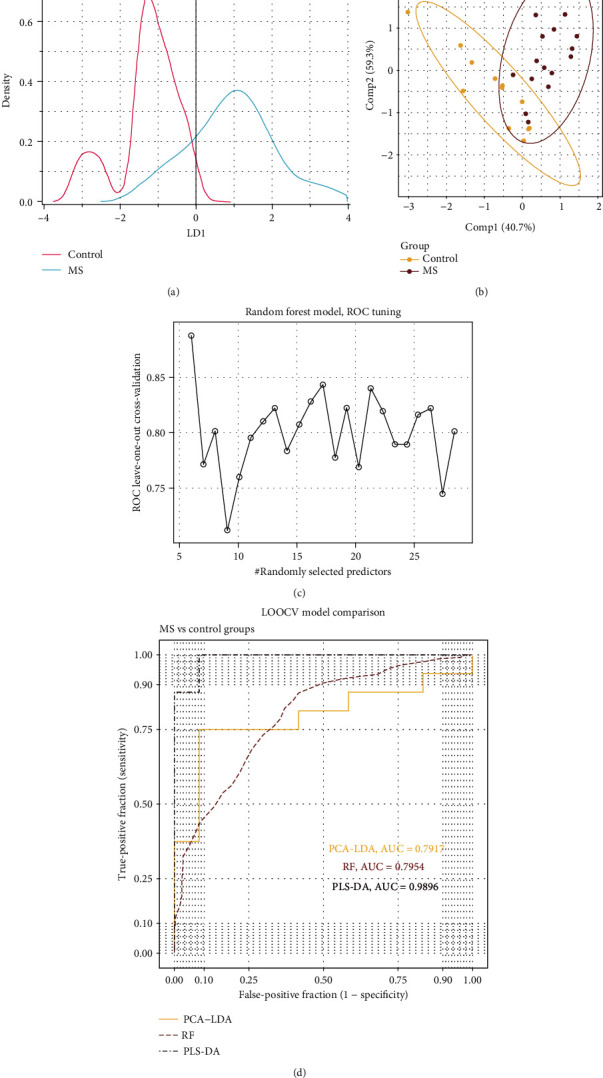
Supervised multivariate analysis of groups “MS” and “control”; ROC of predictive models. (a) LDA with preprocessing by PCA and eight selected principal components. (b) PLS-DA (components 1 and 2). The final model has R2 = 0.79 and Q2 = 0.60. (c) Dependence of the RF ROC value on the number of selected predictors for the 50-tree case model. (d) ROC plots of three predictive models based on multivariate a5nalysis methods: PCA-LDA, RF, and PLS-DA as well as leave-one-out cross-validation.

To carry out supervised LDA, the first eight principal components were chosen. It is remarkable that only the LD1 component was available, and the visualization is depicted in the density plot (Figure 2(a)).

As expected, two groups of observations were well but not perfectly separated by LDA with preprocessing by PCA. Therefore, alternative algorithms were applied to the supervised analysis. Hence, the PLS-DA method (widely used in metabolomics studies) was utilized for data processing [25] (Figure 2(b)). Values for R2 and Q2 were 0.79 and 0.60, respectively. We achieved much better separation of individuals between the two groups in comparison with PCA-LDA. The third algorithm for supervised analysis and predictive model building was RF from the general “caret” package [26]. Optimal tuning parameters (the number of trees equal to 50 and the number of variables in a tree equal to 17) were determined according to the following criterion: a maximum of the sum of three parameters: AUC, sensitivity, and specificity (Figure 2(c)). Predictive models based on the three algorithms were compared by the leave-one-out cross-validation method on a training dataset (Figure 2(d)). The PLS-DA–based model produced the best result on MS prediction with AUC, sensitivity, and specificity of 0.98, 0.81, and 1.0, respectively. PCA-LDA– and RF-based models showed similar predictive effectiveness, with 0.79, 0.67, and 0.75 for the PCA-LDA model and 0.80, 0.64, and 0.80 for the RF model, respectively.

### 3.3. Univariate Statistical Analysis

**Figure 3:**
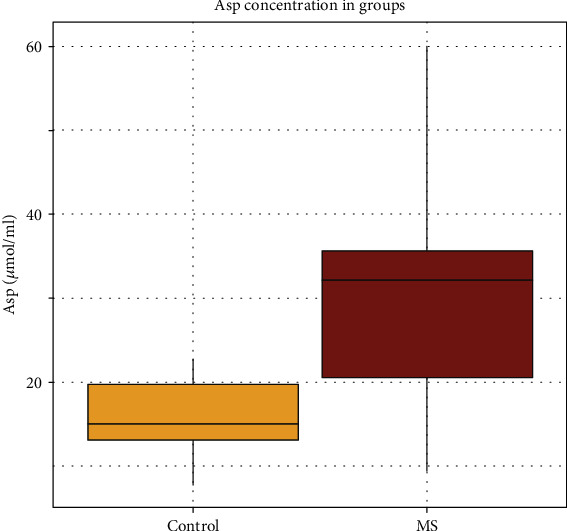
Boxplot interpretation of aspartic acid concentration distribution in the control group and MS group; the bold line indicates a median value, rectangle borders denote an interquartile range, and whiskers represent the minimum and maximum values.

The concentrations measured in dried plasma spots in the control and MS groups are presented in Table 2. According to the Mann–Whitney–Wilcoxon test (Table 2), only aspartic acid levels were significantly different between the two groups (*P* = 0.0097; Figure 3). Mean concentrations and standard deviations of aspartic acid in the plasma samples of the MS group and control group were calculated: 30.65 ± 15.87 and 15.67 ± 5.27 *μ*mol/ml, respectively.

## 4. Discussion

The newly created predictive models are related to the type of classification in machine learning. The goal of this study was to solve two-class classification (MS or control) on the basis of data on the concentrations of amino acids and acylcarnitines in plasma.

Multivariate statistical analysis is ubiquitously used in metabolomics studies and takes advantage of the cumulative power of numerous metabolites for grouping individuals into categories. Predictive models based on the multimarker approach perform well in situations when a single marker is not obvious, but there are many slightly different levels of metabolites between the groups [27]. LDA applied to all variables without a data dimension reduction was not acceptable for our dataset because many variables were collinear (Figure 1(b)). This problem could be overcome by a dimensionality reduction technique, such as PCA or PLS followed by discriminant analysis. In our study, two groups of observations were not separated by components PC1 and PC2 in unsupervised PCA (Figure 1(c)), but they were almost separated by the supervised PLS-DA method (Figure 2(b)). Although we achieved quite good separation by PLS-DA, some scientists believe that the results obtained by the PLS-DA technique tend to be overestimated in some situations [28]. The result obtained by leave-one-out cross-validation via PLS-DA (AUC ≈ 0.98) may not be so optimistic for an independent dataset. The alternative technique we tested was RF, which is widely employed in different areas of machine learning wherever prediction problems need to be solved. Given that this algorithm is quite stable for work with collinear variables, tidy data have been used without preliminary dimension reduction methods such as PCA or PLS [29]. It is noteworthy that the RF model—despite our expectations—was not the most effective (AUC ≈ 0.8), and the result obtained was close to that of the PCA-LDA model (Figure 2(d)).

In our experiment, we used the dried plasma spot method for sample preparation. Some minor acylcarnitines in plasma turned out to be under the threshold (signal/noise < 2); this situation resulted in a reduced number of metabolites available for subsequent statistical analysis and increased the number of missing values in the dataset. It is known that levels of acylcarnitines in plasma and blood are different [30]; consequently, the application of dried blood spots may give a different set of metabolites suitable for statistical analysis.

Glutamic acid and N-acetyl-aspartate (NAA) levels both in CSF and serum are known to be higher in MS [31, 32]. Moreover, NAA is reported to be a specific marker distinguishing MS from neuromyelitis optica [32]. We uncovered only one metabolite (aspartic acid, *P* = 0.0097) whose concentration was substantially different between the groups according to the Mann–Whitney–Wilcoxon test. It was previously determined that the levels of asparagine and glutamate are higher in plasma samples from patients with MS because of activation of the asparagine biosynthesis pathway [19]. In our study, we also observed an increase in the glutamate concentration in the MS group although without significance (*P* = 0.1457; Table 2). Aspartic acid, glutamic acid, and other amino acids are related to the amino acid superpathway, and their metabolism is linked to the tricarboxylic acid cycle and oxaloacetate and *α*-ketoglutarate intermediates; furthermore, aspartic acid is a precursor of NAA [33, 34].

Acylcarnitines are less studied in the field of MS research than amino acids or phospholipids; nevertheless, one study revealed decreasing levels of acylcarnitines in MS without detailed information on individual metabolites [35]. Although we did not find any acylcarnitine whose level was substantially different between the two groups, overall, the concentrations of most acylcarnitines were lower in the MS group (Table 2). Nevertheless, we believe that a change in the acylcarnitine profile is important for the classification of MS and healthy controls because even small changes in levels of individual metabolites (which are under the threshold of significance individually, *P* > 0.05) may result in a high overall score in the whole profile difference between groups. This principle may improve overall results of the classification models that we examined by means of different multivariate algorithms via a cross-validation procedure even in studies with a small sample size.

## 5. Conclusion

By applying different algorithms of multivariate statistics to the same metabolomics dataset, we successfully distinguished MS samples from healthy controls. This result means that amino acid and acylcarnitine profiles are different between the two groups and could serve as a source of data for the development of diagnostic decision support systems. The PLS-DA technique yielded the best classification solution in our study as compared to RF and PCA-LDA algorithms when applied to the same cleaned and scaled data. Aspartic acid levels in plasma were found to be considerably different between MS patients and healthy controls; this preliminary result obtained by comparison of small groups needs further verification.

## Figures and Tables

**Table 1:** Age distribution in control and MS groups.

Group	Min	1^st^ Qu.	Median	Mean	3^rd^ Qu.	Max
Control	23.00	24.00	29.50	29.83	35.25	38.00
MS	22.00	22.00	31.50	30.12	36.25	37.00

**Table 2:** Measured concentrations of metabolites in control and multiple sclerosis groups and Mann–Whitney *U* test comparison for median.

Metabolites	Control group			MS group			M.-W. *U* test *P* value
	Median conc. (*μ*mol/ml)	Mean conc. (*μ*mol/ml)	s.d.	Median conc. (*μ*mol/ml)	Mean conc. (*μ*mol/ml)	s.d.	
Asp	15.0507	15.6657	5.2672	32.1554	30.6449	15.8740	0.0097
C8:1-carnitine	0.0472	0.0494	0.0121	0.0393	0.0407	0.0076	0.0530
C5OH-carnitine	0.0125	0.0125	0.0025	0.0141	0.0154	0.0066	0.0993
Glu	63.2383	62.1980	12.4526	80.8461	89.0735	41.6262	0.1457
C5-carnitine	0.0391	0.0499	0.0353	0.0337	0.0372	0.0226	0.1736
Tyr	44.6430	53.7064	25.8724	37.7501	41.5309	12.5512	0.1736
Val	78.0683	84.5595	30.3860	68.6876	74.3788	18.9949	0.2053
C3-carnitine	0.1186	0.1470	0.0707	0.1117	0.1166	0.0658	0.2226
Cit	18.9037	18.3220	4.0953	16.2658	16.6069	3.5469	0.2601
C8-carnitine	0.0251	0.0404	0.0346	0.0433	0.0416	0.0157	0.2750
Arg	45.6852	45.5796	9.9030	40.7909	41.8867	13.8405	0.3015
Pro	136.2588	160.0983	66.9696	117.1770	138.5637	66.4677	0.3470
C3DC-carnitine	0.0462	0.0449	0.0104	0.0484	0.0498	0.0102	0.3713
Met	4.2999	5.0050	1.9571	3.7745	4.7199	1.9774	0.4228
Phe	27.6062	28.6280	8.4151	22.7213	26.5824	9.0137	0.4228
Leu+Ile	75.4267	82.0059	30.8033	70.1791	75.0415	21.4271	0.5070
Orn	38.8374	39.2365	6.9759	35.1740	38.7100	14.2728	0.5369
C18:1-carnitine	0.1713	0.1752	0.0713	0.1843	0.1804	0.0523	0.5369
C10:1-carnitine	0.0498	0.0710	0.0446	0.0646	0.0610	0.0320	0.6642
Ala	122.9021	113.9486	35.2646	118.6330	124.2386	64.5191	0.8017
C2-carnitine	1.9548	2.3722	0.8908	2.2165	2.3927	0.8565	0.8017
C10-carnitine	0.0442	0.0669	0.0535	0.0543	0.0509	0.0248	0.8017
C4OH-carnitine	0.0128	0.0130	0.0057	0.0127	0.0127	0.0047	0.8344
Carnitine	15.0021	15.3318	4.0812	14.1629	15.7528	7.6604	0.8372
C4-carnitine	0.0572	0.1013	0.0848	0.0634	0.0694	0.0274	0.8731
C14:1-carnitine	0.0165	0.0183	0.0103	0.0176	0.0176	0.0058	0.9445
Gly	98.6914	103.7987	30.5344	101.8677	104.9996	30.7912	0.9454
C6-carnitine	0.0163	0.0278	0.0229	0.0208	0.0208	0.0076	0.9815

s.d.: standard deviation.

## Data Availability

Datasets generated during LC-MS/MS analysis in Wiff format are available from the corresponding author on reasonable request. Preprocessed data for further data analysis in R is available in a Microsoft Excel spreadsheet added to the Figshare data repository [23]. The code reproducing the results of the study from a Microsoft Excel spreadsheet is uploaded to the Github repository (https://github.com/MaratKasakin/MultipleSclerosis).

## Supplementary Materials

Supplementary Materials Supplementary Data 1: the document contains data acquisition parameters from Analyst 1.6.2 Software report about a multiple reaction monitoring method developed on the tandem mass-spectrometer API 3200QTRAP coupled with a Shimadzu Prominence system (autosampler SIL-20AC, controller CBM-20A Lite, and pumps LC-20AD) to collect raw data. Supplementary Table S1: metabolites and stable isotope-labeled internal standards and MRM transitions for derivatives.
