# Supplementary material for: Changes in Amino Acid and Acylcarnitine Plasma Profiles for Distinguishing Patients with Multiple Sclerosis from Healthy Controls
Source: Mult Scler Int. 2020 Jul 15;2020:9010937. doi: 10.1155/2020/9010937 (PMC7378614; doi:10.1155/2020/9010937)
Supplement: Supplementary Materials — Supplementary Data 1: the document contains data acquisition parameters from Analyst 1.6.2 Software report about a multiple reaction monitoring method developed on the tandem mass-spectrometer API 3200QTRAP coupled with a Shimadzu Prominence system (autosampler SIL-20AC, controller CBM-20A Lite, and pumps LC-20AD) to collect raw data. Supplementary Table S1: metabolites and stable isotope-labeled internal standards and MRM transitions for derivatives. [file 9010937.f1.pdf]

Supplementary Data 1 Data acquisition parameters from Analyst 1.6.2 Software report

File Information for Sample 221 (MSc-13-L1(1)) of 2018-04-19.wiff

File Name: 2018-04-19.wiff
File Path: D:\Analyst Data\Projects\Muliple sclerosis\Sclerosis\Data\ Original Name: 2018-04-19.wiff
Software Version: Analyst 1.6.2

Log Information from Devices at Start of acquisition:

Software Application Shimadzu Instrument driver 0

Time from start =0.0000 min Mass Spectrometer 3200 Q TRAP Config Table Version 02
Firmware Version MIA3000--------MIL3002 MIB3000
Component Name Linear Ion Trap Quadrupole LC/MS/MS Mass Spectrometer Component ID 3200 Q TRAP
Manufacturer AB Sciex Instruments
Model 1031491U
Serial Number AF26401304

Time from start =0.0000 min Mass Spectrometer 3200 Q TRAP Start of Run - Detailed Status

Vacuum Status	At Pressure
Vacuum Gauge (10e-5 Torr)	2.9
Backing Pump	Ok
Dual Turbo Pump	Normal
Sample Introduction Status	Ready
Source/Ion Path Electronics	On
Source Type	Turbo Spray
Source Temperature (at setpoint)	300.0 C
Source Exhaust Pump	Ok
Interface Heater	Ready

Time from start =0.0000 min Shimadzu Instrument driver Injection Volume 14 uL
System Controller (CBM-20A Lite) Power On On
Event 1 On
Event 2 Off

Pumps (LC-20AD/LC-20AD)

Pumping Mode	Binary
Flow Total Flow mL/min	0.01
B Concentration	80 %
B Curve	0
P.Min 1	0 MPa
P.Max 1	20 MPa

Pump A

Pump B

Autosampler (SIL-20AC)

Used	Yes
Rinsing Volume	450 uL
Needle Stroke	52 mm
Rinsing Speed	35 uL/sec
Sampling Speed	15 uL/sec
Purge Time	25 min
Dip Time	0 sec
Rinse Mode	Before and after aspiration
Cooler Temperature	10 C
Control Vial Stroke	52 mm

Time Program

Time (min)	Module	Event	Parameter
0.01	Pumps	Pump B Conc.	80
0.01	Pumps	Total Flow	0.1
0.01	System Controller	Event	0
0.6	Pumps	Total Flow	0.1
0.8	Pumps	Total Flow	0.07
1.5	Pumps	Total Flow	0.07
1.51	Pumps	Total Flow	0.4
1.7	Pumps	Total Flow	0.4
1.71	Pumps	Total Flow	0.1
1.75	System Contr		
oller	Stop		

Mass Spectrometer	3200 Q TRAP	0
End of Run - Detailed Status		
Vacuum Status		At Pressure
Vacuum Gauge (10e-5 Torr)		2.9
Backing Pump		Ok
Dual Turbo Pump		Normal
Sample Introduction Status		Ready
Source/Ion Path Electronics		On
Source Type		Turbo Spray
Source Temperature (at setpoint)		300.0 C
Source Exhaust Pump		Ok
Interface Heater		Ready

Time from start =2.1500 min

Acquisition Info

Acquisition Method: \Newborn_amino_acyl_allMRM.dam

Acquisition Path: D:\Analyst Data\Projects\Muliple sclerosis\Sclerosis\Acquisition Methods\

First Sample Started:	Thursday, April 19, 2018 17:10:20
Last Sample Finished:	Friday, April 20, 2018 04:41:47
Sample Acq Time:	Friday, April 20, 2018 03:53:18
Sample Acq Duration:	1min44sec
Number of Scans:	0
Periods in File:	1
Batch Name:	\MSc-13.dab
Batch Path:	D:\Analyst Data\Projects\Muliple sclerosis\Sclerosis\Batch\
Submitted by:	3200QTRAP\Administrator()
Logged-on User:	3200QTRAP\Administrator
Synchronization Mode:	LC Sync
Auto-Equilibration:	Off
Comment:	
Software Version:	Analyst 1.6.2
Set Name:	2018-04-19
Sample Name	MSc-13-L1(1)
Sample ID	
Sample Comments:	
Autosampler Vial:	45
Rack Code:	1.5mL Standard
Rack Position:	1
Plate Code:	1.5mL Standard
Plate Position	1

Valco Valve	Diverter	
	Total Time (min)	Position

Software Application Properties

Display Name:	Shimadzu Instrument driver
Identifier Key:	{51CF5EC0-5D0A-4127-9F32-8128E5B4BF0B}
Method Filename:	None

Method Data:

System Controller

Power On:	On
Event 1:	On
Event 2:	Off

Time Program

Time	Module	Event	Event String
----------	----------	----------	----------
0.01	Pumps	7	Pump B Conc.

0.01	Pumps	6	Total Flow
0.01	System Controller	0	Event
0.6	Pumps	6	Total Flow
0.8	Pumps	6	Total Flow
1.5	Pumps	6	Total Flow
1.51	Pumps	6	Total Flow
1.7	Pumps	6	Total Flow
1.71	Pumps	6	Total Flow
1.75	System Controller	3	Stop

Pumps

Pumping Mode:	Binary Flow
P.Min 1:	0 MPa
P.Max 1:	20 MPa
Total Flow:	0.01 mL/min
B Concentration:	80 %
B Curve:	0

Pump A

Pump B

Autosampler

Used:	Yes
Rinsing Volume:	450 uL
Needle Stroke:	52 mm
Rinsing Speed:	35 uL/sec
Sampling Speed:	15 uL/sec
Purge Time:	25 min
Dip Time:	0 sec
Rinse Mode:	Before and after aspiration
Enable Cooler:	Yes
Cooler Temperature:	10 C
Control Vial Stroke:	52 mm

Quantitation Information:

Sample Type:	Unknown
Dilution Factor:	1.000000

Custom Data:

Quantitation Table:

Period 1:

Scans in Period: 37
Relative Start Time: 0.00 msec
Experiments in Period: 1

Period 1 Experiment 1:

Scan Type: MRM (MRM)
Scheduled MRM: No
Polarity: Positive
Scan Mode: N/A
Ion Source: Turbo Spray
Resolution Q1: Unit
Resolution Q3: Unit
Intensity Thres.: 0.00 cps
Settling Time: 0.0000 msec
MR Pause: 5.0070 msec
MCA: No
Step Size: 0.00 Da

Q1 Mass (Da)	Q3 Mass (Da)	Dwell(msec)	Param	Start
	Stop	ID		
146.100	44.000	35.00	DP	25.00
	25.00	Alanine		
			CEP	10.00
	10.00			
			CE	22.00
	22.00			

Q1 Mass (Da)	Q3 Mass (Da)	Dwell(msec)	Param	Start
	Stop	ID		
150.100	48.000	35.00	DP	25.00
	25.00	Alalnine std		
			CEP	12.00
	12.00			
			CE	22.00
	22.00			

Q1 Mass (Da)	Q3 Mass (Da)	Dwell(msec)	Param	Start
	Stop	ID		
231.100	70.100	35.00	DP	48.00
	48.00	Arginine		

Q1 Mass (Da)	Q3 Mass (Da)	Dwell(msec)	Param	Start	Stop	ID
			CEP	14.00	14.00	
			CE	41.00	41.00	

Q1 Mass (Da)	Q3 Mass (Da)	Dwell(msec)	Param	Start	Stop	ID
238.100	77.100	35.00	DP	48.00	48.00	Arginine std
			CEP	14.00	14.00	
			CE	41.00	41.00	

Q1 Mass (Da)	Q3 Mass (Da)	Dwell(msec)	Param	Start	Stop	ID
246.200	144.100	35.00	DP	28.00	28.00	Asparagine
			CEP	12.00	12.00	
			CE	26.00	26.00	

Q1 Mass (Da)	Q3 Mass (Da)	Dwell(msec)	Param	Start	Stop	ID
249.200	147.100	35.00	DP	28.00	28.00	Asparagine std
			CEP	12.00	12.00	
			CE	26.00	26.00	

Q1 Mass (Da)	Q3 Mass (Da)	Dwell(msec)	Param	Start	Stop	ID
218.200	85.000	35.00	DP	44.00	44.00	C:0
			CEP	12.00	12.00	
			CE	38.00	38.00	

Q1 Mass (Da)	Q3 Mass (Da)	Dwell(msec)	Param	Start
	Stop	ID		
227.200	85.000	35.00	DP	44.00
	44.00	C:0 std		
			CEP	12.00
	12.00			
			CE	38.00
	38.00			

Q1 Mass (Da)	Q3 Mass (Da)	Dwell(msec)	Param	Start
	Stop	ID		
428.400	85.000	35.00	DP	56.00
	56.00	C:14		
			CEP	12.00
	12.00			
			CE	38.00
	38.00			

Q1 Mass (Da)	Q3 Mass (Da)	Dwell(msec)	Param	Start
	Stop	ID		
431.400	85.000	35.00	DP	56.00
	56.00	C14 std		
			CEP	12.00
	12.00			
			CE	38.00
	38.00			

Q1 Mass (Da)	Q3 Mass (Da)	Dwell(msec)	Param	Start
	Stop	ID		
456.400	85.000	35.00	DP	60.00
	60.00	C16		
			CEP	12.00
	12.00			
			CE	38.00
	38.00			

Q1 Mass (Da)	Q3 Mass (Da)	Dwell(msec)	Param	Start
	Stop	ID		

459.400	85.000	35.00	DP	60.00
	60.00	C16 std		
			CEP	12.00
	12.00			
			CE	38.00
	38.00			

Q1 Mass (Da)	Q3 Mass (Da) Stop	Dwell(msec) ID	Param	Start
472.400	85.000	35.00	DP	60.00
	60.00	C16_OH		
			CEP	12.00
	12.00			
			CE	38.00
	38.00			

Q1 Mass (Da)	Q3 Mass (Da) Stop	Dwell(msec) ID	Param	Start
260.200	85.000	35.00	DP	36.00
	36.00	C2		
			CEP	12.00
	12.00			
			CE	38.00
	38.00			

Q1 Mass (Da)	Q3 Mass (Da) Stop	Dwell(msec) ID	Param	Start
263.200	85.000	35.00	DP	36.00
	36.00	C2 std		
			CEP	12.00
	12.00			
			CE	38.00
	38.00			

Q1 Mass (Da)	Q3 Mass (Da) Stop	Dwell(msec) ID	Param	Start
274.200	85.000	35.00	DP	56.00
	56.00	C3		
			CEP	12.00
	12.00			

CE 38.00
38.00

Q1 Mass (Da) Q3 Mass (Da) Dwell(msec) Param Start
Stop ID
277.200 85.000 35.00 DP 56.00
56.00 C3 std
CEP 12.00
12.00
CE 38.00
38.00

Q1 Mass (Da) Q3 Mass (Da) Dwell(msec) Param Start
Stop ID
288.200 85.000 35.00 DP 28.00
28.00 C4
CEP 12.00
12.00
CE 38.00
38.00

Q1 Mass (Da) Q3 Mass (Da) Dwell(msec) Param Start
Stop ID
291.200 85.000 35.00 DP 28.00
28.00 C4 std
CEP 12.00
12.00
CE 38.00
38.00

Q1 Mass (Da) Q3 Mass (Da) Dwell(msec) Param Start
Stop ID
302.200 85.000 35.00 DP 44.00
44.00 C5
CEP 12.00
12.00
CE 38.00
38.00

Q1 Mass (Da)	Q3 Mass (Da)	Dwell(msec)	Param	Start	Stop	ID
311.200	85.000	35.00	DP	44.00	44.00	C5 std
			CEP	12.00	12.00	
			CE	38.00	38.00	

Q1 Mass (Da)	Q3 Mass (Da)	Dwell(msec)	Param	Start	Stop	ID
388.300	85.000	35.00	DP	44.00	44.00	C5_DC
			CEP	12.00	12.00	
			CE	38.00	38.00	

Q1 Mass (Da)	Q3 Mass (Da)	Dwell(msec)	Param	Start	Stop	ID
344.300	85.000	35.00	DP	44.00	44.00	C8
			CEP	12.00	12.00	
			CE	38.00	38.00	

Q1 Mass (Da)	Q3 Mass (Da)	Dwell(msec)	Param	Start	Stop	ID
347.300	85.000	35.00	DP	44.00	44.00	C8 std
			CEP	12.00	12.00	
			CE	38.00	38.00	

Q1 Mass (Da)	Q3 Mass (Da)	Dwell(msec)	Param	Start	Stop	ID
232.100	113.000	35.00	DP	36.00	36.00	Cit

CEP 14.00
14.00
CE 25.00
25.00

Q1 Mass (Da)	Q3 Mass (Da)	Dwell(msec)	Param	Start
	Stop	ID		
234.100	115.000	35.00	DP	36.00
	36.00	Cit_istd		
			CEP	14.00
	14.00			
			CE	25.00
	25.00			

Q1 Mass (Da)	Q3 Mass (Da)	Dwell(msec)	Param	Start
	Stop	ID		
260.200	158.000	35.00	DP	36.00
	36.00	Glu		
			CEP	12.00
	12.00			
			CE	26.00
	26.00			

Q1 Mass (Da)	Q3 Mass (Da)	Dwell(msec)	Param	Start
	Stop	ID		
265.200	163.100	35.00	DP	36.00
	36.00	Glu_std		
			CEP	12.00
	12.00			
			CE	26.00
	26.00			

Q1 Mass (Da)	Q3 Mass (Da)	Dwell(msec)	Param	Start
	Stop	ID		
132.100	76.100	35.00	DP	26.00
	26.00	Gly		
			CEP	12.00
	12.00			
			CE	14.00
	14.00			

Q1 Mass (Da)	Q3 Mass (Da)	Dwell(msec)	Param	Start	Stop	ID
135.100	79.100	35.00	DP	26.00	26.00	Gly std
			CEP	12.00	12.00	
			CE	14.00	14.00	

Q1 Mass (Da)	Q3 Mass (Da)	Dwell(msec)	Param	Start	Stop	ID
206.200	104.100	35.00	DP	28.00	28.00	Met
			CEP	12.00	12.00	
			CE	26.00	26.00	

Q1 Mass (Da)	Q3 Mass (Da)	Dwell(msec)	Param	Start	Stop	ID
209.200	107.100	35.00	DP	28.00	28.00	Met STD
			CEP	12.00	12.00	
			CE	26.00	26.00	

Q1 Mass (Da)	Q3 Mass (Da)	Dwell(msec)	Param	Start	Stop	ID
189.200	70.100	35.00	DP	31.00	31.00	Orn
			CEP	12.00	12.00	
			CE	31.00	31.00	

Q1 Mass (Da)	Q3 Mass (Da)	Dwell(msec)	Param	Start	Stop	ID

195.200	76.100	35.00	DP	31.00
	31.00	Orn STD		
			CEP	12.00
	12.00			
			CE	31.00
	31.00			

Q1 Mass (Da)	Q3 Mass (Da)	Dwell(msec)	Param	Start
	Stop	ID		
222.200	119.900	35.00	DP	28.00
	28.00	Phe		
			CEP	12.00
	12.00			
			CE	26.00
	26.00			

Q1 Mass (Da)	Q3 Mass (Da)	Dwell(msec)	Param	Start
	Stop	ID		
227.200	125.100	35.00	DP	28.00
	28.00	Phe STD		
			CEP	12.00
	12.00			
			CE	26.00
	26.00			

Q1 Mass (Da)	Q3 Mass (Da)	Dwell(msec)	Param	Start
	Stop	ID		
238.200	136.100	35.00	DP	28.00
	28.00	Tyr		
			CEP	12.00
	12.00			
			CE	26.00
	26.00			

Q1 Mass (Da)	Q3 Mass (Da)	Dwell(msec)	Param	Start
	Stop	ID		
242.200	140.100	35.00	DP	28.00
	28.00	Tyr STD		
			CEP	12.00
	12.00			

			CE	26.00
	26.00			

Q1 Mass (Da)	Q3 Mass (Da)	Dwell(msec)	Param	Start
	Stop	ID		
174.200	72.100	35.00	DP	28.00
	28.00	Val		
			CEP	12.00
	12.00			
			CE	26.00
	26.00			

Q1 Mass (Da)	Q3 Mass (Da)	Dwell(msec)	Param	Start
	Stop	ID		
182.200	80.100	35.00	DP	28.00
	28.00	Val STD		
			CEP	12.00
	12.00			
			CE	26.00
	26.00			

Q1 Mass (Da)	Q3 Mass (Da)	Dwell(msec)	Param	Start
	Stop	ID		
188.200	86.000	35.00	DP	28.00
	28.00	ile		
			CEP	12.00
	12.00			
			CE	26.00
	26.00			

Q1 Mass (Da)	Q3 Mass (Da)	Dwell(msec)	Param	Start
	Stop	ID		
191.200	89.000	35.00	DP	28.00
	28.00	ile STD		
			CEP	12.00
	12.00			
			CE	26.00
	26.00			

Q1 Mass (Da)	Q3 Mass (Da)	Dwell(msec)	Param	Start
	Stop	ID		
394.300	85.000	35.00	DP	44.00
	44.00	C5DC STD		
			CEP	12.00
	12.00			
			CE	38.00
	38.00			

Q1 Mass (Da)	Q3 Mass (Da)	Dwell(msec)	Param	Start
	Stop	ID		
316.300	85.000	35.00	DP	44.00
	44.00	C6		
			CEP	12.00
	12.00			
			CE	38.00
	38.00			

Q1 Mass (Da)	Q3 Mass (Da)	Dwell(msec)	Param	Start
	Stop	ID		
319.300	85.000	35.00	DP	44.00
	44.00	C6 STD		
			CEP	12.00
	12.00			
			CE	38.00
	38.00			

Q1 Mass (Da)	Q3 Mass (Da)	Dwell(msec)	Param	Start
	Stop	ID		
372.400	85.000	35.00	DP	48.00
	48.00	C10		
			CEP	12.00
	12.00			
			CE	38.00
	38.00			

Q1 Mass (Da)	Q3 Mass (Da)	Dwell(msec)	Param	Start
	Stop	ID		
375.400	85.000	35.00	DP	48.00
	48.00	C10 STD		

	12.00		CEP	12.00
	38.00		CE	38.00

Q1 Mass (Da)	Q3 Mass (Da) Stop	Dwell(msec) ID	Param	Start
400.400	85.000 52.00	35.00 C12	DP	52.00
	12.00		CEP	12.00
	38.00		CE	38.00

Q1 Mass (Da)	Q3 Mass (Da) Stop	Dwell(msec) ID	Param	Start
403.400	85.000 52.00	35.00 C12 STD	DP	52.00
	12.00		CEP	12.00
	38.00		CE	38.00

Q1 Mass (Da)	Q3 Mass (Da) Stop	Dwell(msec) ID	Param	Start
484.400	85.000 60.00	35.00 C18	DP	60.00
	12.00		CEP	12.00
	43.00		CE	43.00

Q1 Mass (Da)	Q3 Mass (Da) Stop	Dwell(msec) ID	Param	Start
487.400	85.000 60.00	35.00 C18 STD	DP	60.00
	12.00		CEP	12.00
	43.00		CE	43.00

Q1 Mass (Da)	Q3 Mass (Da)	Dwell(msec)	Param	Start
	Stop	ID		
318.400	85.000	35.00	DP	44.00
	44.00	C5OH		
			CEP	12.00
	12.00			
			CE	38.00
	38.00			

Q1 Mass (Da)	Q3 Mass (Da)	Dwell(msec)	Param	Start
	Stop	ID		
300.300	85.000	35.00	DP	44.00
	44.00	C5:1		
			CEP	12.00
	12.00			
			CE	38.00
	38.00			

Q1 Mass (Da)	Q3 Mass (Da)	Dwell(msec)	Param	Start
	Stop	ID		
374.300	85.000	35.00	DP	44.00
	44.00	C4DC		
			CEP	12.00
	12.00			
			CE	38.00
	38.00			

Q1 Mass (Da)	Q3 Mass (Da)	Dwell(msec)	Param	Start
	Stop	ID		
482.400	85.000	35.00	DP	60.00
	60.00	C18:1		
			CEP	51.37
	51.37			
			CE	38.00
	38.00			

Q1 Mass (Da)	Q3 Mass (Da)	Dwell(msec)	Param	Start
	Stop	ID		

426.400	85.000	35.00	DP	56.00	56.00	C14:1
			CEP	12.00	12.00	
			CE	38.00	38.00	

Q1 Mass (Da)	Q3 Mass (Da)	Dwell(msec)	Param	Start	Stop	ID
360.300	85.000	35.00	DP	44.00	44.00	C3DC
			CEP	12.00	12.00	
			CE	38.00	38.00	

Q1 Mass (Da)	Q3 Mass (Da)	Dwell(msec)	Param	Start	Stop	ID
304.300	85.000	35.00	DP	44.00	44.00	C4OH
			CEP	12.00	12.00	
			CE	38.00	38.00	

Q1 Mass (Da)	Q3 Mass (Da)	Dwell(msec)	Param	Start	Stop	ID
342.300	85.000	35.00	DP	44.00	44.00	C8:1
			CEP	12.00	12.00	
			CE	38.00	38.00	

Q1 Mass (Da)	Q3 Mass (Da)	Dwell(msec)	Param	Start	Stop	ID
370.400	85.000	35.00	DP	48.00	48.00	C10:1
			CEP	12.00	12.00	

			CE	38.00
	38.00			

Q1 Mass (Da)	Q3 Mass (Da) Stop	Dwell(msec) ID	Param	Start
424.400	85.000	35.00	DP	56.00
	56.00	C14:2		
			CEP	12.00
	12.00			
			CE	38.00
	38.00			

Q1 Mass (Da)	Q3 Mass (Da) Stop	Dwell(msec) ID	Param	Start
444.400	85.000	35.00	DP	60.00
	60.00	C14OH		
			CEP	12.00
	12.00			
			CE	38.00
	38.00			

Q1 Mass (Da)	Q3 Mass (Da) Stop	Dwell(msec) ID	Param	Start
454.400	85.000	35.00	DP	60.00
	60.00	C16:1		
			CEP	12.00
	12.00			
			CE	38.00
	38.00			

Q1 Mass (Da)	Q3 Mass (Da) Stop	Dwell(msec) ID	Param	Start
470.400	85.000	35.00	DP	60.00
	60.00	C16:1OH		
			CEP	12.00
	12.00			
			CE	38.00
	38.00			

Q1 Mass (Da)	Q3 Mass (Da)	Dwell(msec)	Param	Start	Stop	ID
498.400	85.000	35.00	DP	60.00	60.00	C18:1OH
			CEP	12.00	12.00	
			CE	38.00	38.00	

Q1 Mass (Da)	Q3 Mass (Da)	Dwell(msec)	Param	Start	Stop	ID
496.400	85.000	35.00	DP	60.00	60.00	C18:2OH
			CEP	12.00	12.00	
			CE	38.00	38.00	

Q1 Mass (Da)	Q3 Mass (Da)	Dwell(msec)	Param	Start	Stop	ID
500.400	85.000	35.00	DP	60.00	60.00	C18OH
			CEP	12.00	12.00	
			CE	38.00	38.00	

Q1 Mass (Da)	Q3 Mass (Da)	Dwell(msec)	Param	Start	Stop	ID
172.000	116.000	35.00	DP	24.00	24.00	Pro
			CEP	12.00	12.00	
			CE	13.00	13.00	

Q1 Mass (Da)	Q3 Mass (Da)	Dwell(msec)	Param	Start	Stop	ID
179.000	123.000	35.00	DP	24.00	24.00	Pro STD

			CEP	12.00
	12.00			
			CE	13.00
	13.00			

Q1 Mass (Da)	Q3 Mass (Da) Stop	Dwell(msec) ID	Param	Start
461.600	85.000 60.00	35.00 Unknown	DP	60.00
			CEP	50.32
	50.32			
			CE	38.00
	38.00			

Parameter Table(Period 1 Experiment 1)

CUR:	20.00
CAD:	Medium
IS:	5000.00
TEM:	300.00
GS1:	30.00
GS2:	30.00
ihe:	ON
EP	10.00
CXP	4.00

Resolution tables

Quad 1 Positive Unit
Last Modification Date Time: March 23, 2017 13:59:06

IE1 0.800

Mass (Da)	Offset Value
59.050	0.050
175.133	0.122
616.464	0.365
906.673	0.525
1254.925	0.679
1545.134	0.811

Quad 3 Positive Unit
Last Modification Date Time: April 19, 2018 16:27:42

IE3 2.800

Mass (Da)	Offset Value
59.050	0.033

175.133	0.085
616.464	0.170
906.673	0.293
1254.925	0.345
1545.134	0.305

Calibration tables

Quad 1 Positive Unit Resolution
Last Modification Date Time: April 19, 2018 16:30:07

Mass (Da)	Dac Value
59.050	1848
175.133	5549
616.464	19629
906.673	28893
1254.925	39996
1545.134	49249

Quad 3 Positive Unit Resolution
Last Modification Date Time: April 19, 2018 16:30:50

Mass (Da)	Dac Value
59.050	1848
175.133	5561
616.464	19682
906.673	28979
1254.925	40100
1545.134	49380

Instrument Parameters:

Detector Parameters (Positive):
CEM 2450.0

Keyed Text:

File was created with the software version: Analyst 1.6.2

Supplementary Table S1 Metabolites and stable isotope-labeled internal standards, MRM transitions for derivatives

Precursor ion	Product ion	AA/IS
146,1	44	Alanine
150,1	48	Alalnine-D4
231,1	70,1	Arginine
238,1	77,1	Arginine-D7
246,2	144,1	Aspartic acid
249,2	147,1	Aspartic acid-D7
232,1	113	Cit
234,1	115	Cit-D2
260,2	158	Glu
265,2	163,1	Glu-D5
132,1	76,1	Gly
135,1	79,1	Gly-13C2-15N1
206,2	104,1	Met
209,2	107,1	Met-D3
189,2	70,1	Orn
195,2	76,1	Orn-D6
222,2	119,9	Phe
227,2	125,1	Phe-D5
238,2	136,1	Tyr
242,2	140,1	Tyr-D4
174,2	72,1	Val
182,2	80,1	Val-D8
188,2	86	xle
191,2	89	Leu-D3
172	116	Pro
179	123	Pro-D7
218,2	85	C:0
227,2	85	C:0-D9

Precursor ion	Product ion	AC/IS	Compound Name
260,2	85	C2	Acetyl-L-carnitine
263,2	85	C2-D3	
274,2	85	C3	Propionyl-L-carnitine
360,3	85	C3DC	Malonyl-L-carnitine
277,2	85	C3-D3	
288,2	85	C4	Butyryl-L-carnitine
374,3	85	C4DC	Succinyl-L-carnitine
304,3	85	C4OH	Hydroxybutyryl-L-carnitine
291,2	85	C4-D3	
302,2	85	C5	Valeryl-L-carnitine
318,4	85	C5OH	Hydroxyvaleryl-L-carnitine
300,3	85	C5:1	Tiglyl-L-carnitine
311,2	85	C5-D9	
388,3	85	C5_DC	Glutaryl-L-carnitine
394,3	85	C5DC-D6	
316,3	85	C6	Hexanoyl-L-carnitine
319,3	85	C6-D3	
344,3	85	C8	Octanoyl-L-carnitine
342,3	85	C8:1	Octenoyl-L-carnitine
347,3	85	C8-D3	
372,4	85	C10	Decanoyl-L-carnitine
370,4	85	C10:1	Decenoyl-L-carnitine
375,4	85	C10-D3	
400,4	85	C12	Dodecanoyl-L-carnitine
403,4	85	C12-D3	
428,4	85	C14	Tetradecanoyl-L-carnitine
426,4	85	C14:1	Tetradecenoyl-L-carnitine
424,4	85	C14:2	Tetradecadienyl-L-carnitine
444,4	85	C14OH	Hydroxytetradecanoyl-L-carnitine
431,4	85	C14-D3	
456,4	85	C16	Hexadecanoyl-L-carnitine
472,4	85	C16OH	Hydroxyhexadecanoyl-L-carnitine
454,4	85	C16:1	Hexadecenoyl-L-carnitine
470,4	85	C16:1OH	Hydroxyhexadecenoyl-L-carnitine
459,4	85	C16-D3	
484,4	85	C18	Octadecanoyl-L-carnitine
482,4	85	C18:1	Octadecenoyl-L-carnitine
498,4	85	C18:1OH	Hydroxyoctadecenoyl-L-carnitine
496,4	85	C18:2OH	Hydroxyoctadecadienyl-L-carnitine
500,4	85	C18OH	Hydroxyoctadecanoyl-L-carnitine
487,4	85	C18-D3	
